# Osteocutaneous-flap-related osteomyelitis following mandibular reconstruction: a cohort study of an emerging and complex bone infection

**DOI:** 10.5194/jbji-7-127-2022

**Published:** 2022-06-10

**Authors:** Clément Javaux, Clémentine Daveau, Clotilde Bettinger, Mathieu Daurade, Céline Dupieux-Chabert, Fabien Craighero, Carine Fuchsmann, Philippe Céruse, Arnaud Gleizal, Nicolas Sigaux, Tristan Ferry, Florent Valour, the Lyon BJI study group

**Affiliations:** 1 Department of Infectious Diseases, Groupement Hospitalier Nord, Hospices Civils de Lyon, Lyon, 69004, France; 2 Regional Reference Center for the Management of Complex Bone and Joint Infections, Hospices Civils de Lyon, Lyon, 69004, France; 3 Department of Otolaryngology Surgery, Groupement Hospitalier Nord, Hospices Civils de Lyon, Lyon, 69004, France; 4 Department of Anesthesiology, Groupement Hospitalier Nord, Hospices Civils de Lyon, Lyon, 69004, France; 5 Department of Oral and Maxillofacial Surgery, Groupement Hospitalier Nord, Hospices Civils de Lyon, Lyon, 69004, France; 6 Laboratory of Bacteriology, Institute of Infectious Agents, Groupement Hospitalier Nord, Hospices Civils de Lyon, Lyon, 69004, France; 7 Centre International de Recherche en Infectiologie (CIRI), Inserm, U1111, Université Claude Bernard Lyon 1, CNRS, UMR5308, Ecole Normale Supérieure de Lyon, Lyon, 69007, France; 8 Department of Radiology, Groupement Hospitalier Nord, Hospices Civils de Lyon, Lyon, 69004, France; 9 Department of Oral and Maxillofacial Surgery, Groupement Hospitalier Sud, Hospices Civils de Lyon, Pierre-Bénite, 69310, France; ➕ A full list of authors appears at the end of the paper

## Abstract

Osteocutaneous flap (OCF) mandible reconstruction is at high risk for surgical site infection. This study aimed to describe diagnosis, management, and outcome of OCF-related osteomyelitis. All patients managed at our institution for an OCF-related osteomyelitis following mandible reconstruction were included in a retrospective cohort study (2012–2019). Microbiology was described according to gold-standard surgical samples, considering all virulent pathogens, and potential contaminants if present on at least two samples. Determinants of treatment failure were assessed by logistic regression and Kaplan–Meier curve analysis. The 48 included patients (median age 60.5 (IQR, 52.4–66.6) years) benefited from OCF mandible reconstruction mostly for carcinoma ( n=27/48 ; 56.3 %) or osteoradionecrosis ( n=12/48 ; 25.0 %). OCF-related osteomyelitis was mostly early ( ≤3 months post-surgery; n=43/48 ; 89.6 %), presenting with local inflammation ( n=28/47 ; 59.6 %), nonunion (wound dehiscence) or sinus tract ( n=28/47 ; 59.6 %), and/or bone or device exposure ( n=21/47 ; 44.7 %). Main implicated pathogens were Enterobacteriaceae ( n=25/41 ; 61.0 %), streptococci ( n=22/41 ; 53.7 %), *Staphylococcus aureus* ( n=10/41 ; 24.4 %), enterococci ( n=9/41 ; 22.0 %), non-fermenting Gram-negative bacilli ( n=8/41 ; 19.5 %), and anaerobes ( n=8/41 ; 19.5 %). Thirty-nine patients (81.3 %) benefited from surgery, consisting of debridement with implant retention (DAIR) in 25/39 (64.1 %) cases, associated with 93 (IQR, 64–128) days of antimicrobial therapy. After a follow-up of 18 (IQR, 11–31) months, 24/48 (50.0 %) treatment failures were observed. Determinants of treatment outcomes were DAIR (OR, 3.333; 95 % CI, 1.020–10.898) and an early infectious disease specialist referral (OR, 0.236 if ≤2 weeks; 95 % CI, 0.062–0.933).

OCF-related osteomyelitis following mandibular reconstruction represents difficult-to-treat infections. Our results advocate for a multidisciplinary management, including an early infectious-disease-specialist referral to manage the antimicrobial therapy driven by complex microbiological documentation.

## 1 Introduction

Osteocutaneous flaps (OCF), mostly consisting of free fibular flaps, are increasingly used for mandibular reconstruction after head and neck cancer surgery but also in rarer indications such as benign tumors, mandibular chronic osteomyelitis, radionecrosis/chemonecrosis of the jaw, or posttraumatic reconstruction. This complex technique is associated with a high risk of perioperative complications, reaching 54 % in some studies (Eskander et al., 2018; Suh et al., 2004), and especially a 13 % to 41 % risk of surgical site infection (SSI) (Kamizono et al., 2014; Karakida et al., 2010; Makiguchi et al., 2019; Cannon et al., 2017). This high SSI rate is probably due to the clean-contaminated surgical field with intrabuccal exposure. Furthermore, flap reconstruction gathers many procedure-associated risk factors of SSI – including long surgery duration, blood loss, and use of tracheostomy (Lee et al., 2011; Lin et al., 2018; Mücke et al., 2012) – and permits us to treat more aggressively advanced tumors in a population highly exposed to SSI due to advanced age, frequent co-morbidities, tobacco consumption, and impaired nutritional status (Kamizono et al., 2014; Makiguchi et al., 2019; Lee et al., 2011; Khariwala et al., 2016; Shum et al., 2014). Along with radiotherapy or chemotherapy for cancer management (Bourget et al., 2011; Benatar et al., 2013; Halle et al., 2017), such conditions can lead to infection despite an appropriate antimicrobial prophylaxis (Durand et al., 2015; Haidar et al., 2018). Osteomyelitis following OCF mandible reconstruction is associated with high morbidity and risk of flap failure (Kamizono et al., 2014; Karakida et al., 2010; Makiguchi et al., 2019; Cannon et al., 2017). As described for other postoperative bone and joint infections (BJIs) (Grammatico-Guillon et al., 2012), it may require revision surgery, prolonged broad-spectrum antimicrobial therapy, and extended hospital stay, leading to impaired quality of life and considerable medical expenditure.

Very few studies described mandibular OCF-related osteomyelitis, and there are no specific guidelines for its management. Consequently, practices are highly heterogeneous, often driven by prosthetic joint infection recommendations (Kapadia et al., 2016; ICM, 2022). However, anatomical conditions and pathophysiological pathways of these infections are very different, which should result in specific diagnosis and management considerations.

In this study, we aimed to describe clinical and microbiological diagnostic features, management, and outcome of OCF-related osteomyelitis following mandibular reconstruction in a referral center for the management of complex BJI.

## 2 Patients and methods

### 2.1 Study design and data collection

All adult patients with mandibular OCF-related osteomyelitis followed up in our reference center for the management of complex BJI between 1 September 2012 and 31 July 2019; they were included in a retrospective observational cohort study. Infections requiring less than 1 month of antimicrobial therapy were considered skin and soft tissue infections (SSTIs), so these cases were excluded.

For each patient, data were collected from medical records and biological software in an anonymous standardized case report form. Co-morbidities were summarized by the Charlson co-morbidity index and the American Society of Anesthesiologists (ASA) score.

### 2.2 Surgical procedure

The harvesting step was performed through an anterior or posterior approach, with or without pneumatic tourniquet, depending on the operator's habits, using 2.0 magnifying glasses. A dose of platelet aggregation inhibitor was administered to the patient just before flap ischemia. Perforators were located visually in the distal third of the leg at the beginning of the procedure, with non-routine help of an ultrasound Doppler. The skin paddle was positioned with the most suitable perforator (size, pulsatility) in its center. If possible, two perforators were included. The skin paddle could be set intraorally or extraorally depending on the defect to be reconstructed, without tension on the suture line. If both sides had to be reconstructed, we used the skin paddle for extraoral reconstruction and a muscle for the intraoral side (either the tibialis posterior or a part of the soleus). The donor vessels were the fibular artery which divided just below the bifurcation of the tibiofibular trunk and one of the fibular veins. Arterial anastomoses were performed in a termino-terminal fashion using separate stitches of 8-0 or 9-0 non-absorbable sutures (Ethilon™, Ethicon Inc., Scotland, 1915). Venous anastomoses are either performed in a termino-terminal fashion on one of the proximal branches of the thyro-linguo-facial trunk or in a termino-lateral fashion on the internal jugular vein in second intention, with separate stitches of non-absorbable thread. Regarding bone reconstruction, most of the patients included in the study benefited from preoperative digital planning, custom-made cutting guide, and patient-specific plates with load sharing osteosynthesis. After surgery, flaps were monitored clinically to detect early microvascular failure, using the skin palette as control (heat, color, skin recolouration time). Additionally, Doppler flowmetry monitoring was performed twice daily during the postoperative period, during the entire stay (20 d on average).

### 2.3 Definitions

In the absence of consensual definition of OCF-associated osteomyelitis, diagnosis was based upon clinical, biological, imaging evidence, and/or culture of microorganisms from deep surgical samples, according to the definition of surgical site infection of the US Centers for Disease Control and Prevention (CDC) (Mangram et al., 1999).

On the basis of prosthetic joint infection chronological definitions (Kapadia et al., 2016; ICM, 2022), the delay between inoculation and symptoms was defined as early ( ≤3 months), delayed (3–12 months), and late ( >12 months) postoperative infections. Similarly, infection was classified as “acute” when the delay between inoculation and management was ≤4 weeks.

All intraoperative tissue samples were inoculated onto a Columbia sheep's blood agar plate (read at days 1 and 2), two PolyVitex chocolate agar plates (read at days 1 and 2 for one plate and days 7 and 10 for the other), two blood agar plates for anaerobic incubation (read at days 3 and 5 for one plate and at days 7 and 10 for the other), and into a Schaedler anaerobic liquid broth. The broth was systematically subcultured on day 10 onto chocolate and blood agar plates for anaerobic incubation for 5 d. Identification and antibiotic susceptibility testing were performed following standard laboratory procedures (VITEK 2 system or VITEK MALDI-TOF MS; disk diffusion methods or ATB ANA device; bioMérieux, Marcy l'Etoile, France). Microbiological documentation was described, considering all virulent pathogens (i.e., *Staphylococcus aureus*, streptococci, and Gram-negative bacilli (GNB)) yielded out of deep surgical samples, and low virulent pathogens and/or potential contaminants (i.e., coagulase-negative staphylococci (CoNS), *Corynebacterium* spp., and *Cutibacterium* spp.) if present on at least two deep surgical samples, which is considered the gold standard (Osmon et al., 2013).

Antimicrobial therapy was considered empirical in the absence of previous microbiological documentation. It was considered appropriate when all pathogens highlighted by gold-standard sample cultures were targeted by at least one molecule.

Treatment failure was defined as symptom persistence under treatment (i.e., worsening or recurrence of local inflammatory symptoms, nonunion, sinus tract, abscess, purulent discharge, and bone and/or device exposure), infection relapse after treatment disruption, infection-related requirement of additional surgical procedure, flap loss, and infection-related death.

### 2.4 Statistical analysis

Descriptive statistics were used to estimate the frequencies of the study variables, described as numbers (%) for dichotomous values and medians (interquartile range, IQR) for continuous values. For the percentage calculation of each variable, the number of missing values was excluded from the denominator. Nonparametric statistical methods were used to compare the study groups (chi-squared test, Fisher's exact test, or Mann–Whitney U test, as appropriate). Determinants of treatment failure were assessed using: (i) logistic regression analysis, expressed as odds ratios (OR) and their 95 % confidence intervals (95 % CI), and (ii) Kaplan–Meier curve analysis representing the probability of treatment-failure-free survival with time, compared between groups using the log-rank (Mantel–Cox) test. A p value of ≤0.05 was considered significant. All analyses were performed using SPSS software version 17.0 (SPSS, Chicago, IL, USA) and GraphPad Prism version 5.3 (GraphPad Software, San Diego, CA, USA).

**Table 1:** Description of the included population and comparison of patients with favorable outcome or treatment failure.

	Descriptive analysis				Univariate analysis
	Total population	Favorable	Treatment	P value	OR (95 % CI)
	( n=48 )	outcome ( n=24 )	failure ( n=24 )		
Demographics					
Male, no. (%)	30/48 (62.5 %)	16/24 (66.7 %)	14/24 (58.3 %)	0.551	0.700 (0.216–2.265)
Age, median (IQR), year	60.5 (52.4–66.6)	61.7 (52.2–68.1)	59.6 (52.4–64.2)	0.386	0.826 (0.500–1.363) ${}^{a}$
Co-morbidities					
ASA score, median (IQR)	2 (2.2)	2 (2–2)	2 (2–2.8)	0.374	1.789 (0.617–5.188)
Modified Charlson co-morbidity index, median (IQR)	4 (3–5)	4 (3–5)	3.5 (3–4)	0.126	0.833 (0.624–1.12)
Active tobacco consumption, no. (%)	14/47 (29.8 %)	8/24 (33.3 %)	6/23 (26.1 %)	0.587	0.706 (0.200–2.487)
Underlying mandibular condition					
Carcinoma, no. (%)	27/48 (56.3 %)	11/24 (45.8 %)	16/24 (66.7 %)	0.146	2.364 (0.735–7.603)
Osteoradionecrosis, no. (%)	12/48 (25.0 %)	9/24 (37.5 %)	3/24 (12.5 %)	0.093	0.238 (0.055–1.030)
Osteomyelitis, no. (%)	7/48 (14.6 %)	3/24 (12.5 %)	4/24 (16.7 %)	>0.999	1.44 (0.278–7.056)
Oncologic adjuvant therapies					
Radiotherapy, no. (%)	34/41 (82.9 %)	16/21 (76.2 %)	18/20 (90.0 %)	0.410	2.812 (0.478–16.557)
Chemotherapy, no. (%)	14/27 (51.9 %)	6/11 (54.5 %)	8/16 (50.0 %)	0.816	0.833 (0.179–3.884)
Infection characteristics					
Delay from inoculation to symptoms, median (IQR), week	2.6 (1.0–5.4)	2.0 (0.6–3.9)	3.1 (1.2–5.5)	0.270	1.007 (0.914–1.109)
Early infection ( ≤3 months), no. (%)	43/48 (89.6 %)	21/24 (87.5 %)	22/24 (91.7 %)	>0.999	1.571 (0.238–10.365)
Delayed infection (3–12 months), no. (%)	5/48 (10.4 %)	3/24 (12.5 %)	2/24 (8.3 %)	>0.999	0.636 (0.096–4.197)
Delay from inoculation to surgery, median (IQR), w	8.9 (1.6–27.6)	2.9 (1.2–28.6)	15.9 (2.7–27.4)	0.593	1.013 (0.977–1.050)
Acute infection ( ≤4 weeks), no. (%)	22/48 (45.8 %)	13/24 (54.2 %)	9/24 (37.5 %)	0.247	0.508 (0.160–1.607)
Clinical features					
Fever, no. (%)	17/47 (36.2 %)	7/24 (29.2 %)	10/23 (43.5 %)	0.307	1.868 (0.559–6.240)
Local inflammatory symptoms, no. (%)	28/47 (59.6 %)	15/24 (62.5 %)	13/23 (56.5 %)	0.676	0.780 (0.243–2.506)
Pain, no. (%)	11/47 (23.4 %)	6/24 (25.0 %)	5/23 (21.7 %)	0.792	0.833 (0.215–3.230)
Delayed wound healing, no. (%)	21/47 (44.7 %)	8/24 (33.3 %)	13/23 (56.5 %)	0.110	2.600 (0.796–8.488)
Nonunion/sinus tract, no. (%)	28/47 (59.6 %)	13/24 (54.2 %)	15/23 (65.2 %)	0.440	1.587 (0.490–5.138)
Bone and/or device exposure, no. (%)	21/47 (44.7 %)	9/24 (37.5 %)	12/23 (52.2 %)	0.312	1.818 (0.568–5.817)
Tissue necrosis, no. (%)	15/48 (31.3 %)	5/24 (20.8 %)	10/24 (41.7 %)	0.117	2.614 (0.757–9.727)
Purulent discharge, no. (%)	31/47 (66.0 %)	16/24 (66.7 %)	15/23 (65.2 %)	0.917	0.938 (0.280–3.134)
Abscess, no. (%)	22/47 (46.8 %)	11/24 (45.8 %)	11/23 (47.8 %)	0.891	1.083 (0.344–3.409)
Biological findings					
Maximum CRP level, median (IQR), mg L ${}^{-1}$	90.3 (34.2–158.5)	119.0 (55.2–173.5)	73.2 (25.4–107.0)	0.537	0.993 (0.985–1.002)
Maximum WBC count, median (IQR), G/L	13.1 (10.1–16.3)	13.2 (11.1–16.3)	12.3 (9.8–16.3)	0.773	1.017 (0.897–1.154)
Radiological evaluation, no. (%)	44/48 (91.7 %)	22/24 (91.7 %)	22/24 (95.8 %)	n/a	n/a
Radiological signs for infection, no. (%)	33/44 (75.0 %)	15/22 (68.2 %)	18/22 (81.8 %)	0.488	2.100 (0.514–8.573)
Bone lysis, no. (%)	15/44 (34.1 %)	5/22 (22.7 %)	10/22 (45.5 %)	0.203	2.833 (0.770–10.430)
Bone nonunion/pseudarthrosis, no. (%)	8/44 (18.2 %)	2/22 (9.1 %)	6/22 (27.3 %)	0.240	3.750 (0.665–21.154)
Implant migration/fracture, no. (%)	12/44 (27.3 %)	4/22 (18.2 %)	8/22 (36.4 %)	0.310	2.571 (0.641–10.310)
Abscess, no. (%)	21/44 (47.7 %)	10/22 (45.5 %)	11/22 (50.0 %)	>0.999	1.200 (0.367–3.922)
Microbiological findings (gold standard)					
Gold-standard samples, no. (%)	41/48 (85.4 %)	19/24 (79.2 %)	22/24 (91.7 %)	0.416	2.895 (0.503–16.674)
No. of samples, median (IQR)	3 (1–5)	2 (1–4.5)	3 (2–4.8)	0.810	1.168 (0.897–1.521)
Documented infection, no. (%)	40/41 (97.6 %)	19/19 (100 %)	21/22 (95.5 %)	>0.999	NC
*Staphylococcus aureus*, no. (%)	10/41 (24.4 %)	6/19 (31.6 %)	4/22 (18.2 %)	0.469	0.481 (0.133–2.058)
MRSA, no. (%)	1/41 (2.4 %)	1/19 (5.3 %)	0/22 (0 %)	0.463	NC
CoNS, no. (%)	4/41 (9.8 %)	2/19 (10.5 %)	2/22 (9.1 %)	>0.999	0.850 (0.108–6.695)
MRCoNS, no. (%)	2/41 (4.9 %)	0/19 (0 %)	2/22 (9.1 %)	0.490	NC
*Streptococcus* spp., no. (%)	22/41 (53.7 %)	10/19 (52.6 %)	12/22 (54.5 %)	0.902	1.080 (0.315–3.698)
*Enterococcus* spp., no. (%)	9/41 (22.0 %)	4/19 (21.1 %)	5/22 (22.7 %)	>0.999	1.103 (0.249–4.878)
Enterobacteriaceae, no. (%) ${}^{b}$	25/41 (61.0 %)	12 (63.2 %)	13/22 (59.1 %)	>0.999	0.843 (0.239–2.975)
ESBL-secreting Enterobacteriaceae, no. (%)	3/41 (7.3 %)	1/19 (5.3 %)	2/22 (9.1 %)	>0.999	1.800 (0.150–21.569)
Non-fermenting GNB, no. (%)	8/41 (19.5 %)	1/19 (5.3 %)	7/22 (31.8 %)	0.05	8.400 (0.927–76.151)
*Cutibacterium acnes*, no. (%)	1/41 (2.4 %)	1/19 (5.3 %)	0/22 (0 %)	0.463	NC
*Actinomyces* spp., no. (%)	2/41 (4.9 %)	1/19 (5.3 %)	1/22 (4.5 %)	>0.999	0.857 (0.05–14.706)
Other anaerobes, no. (%)	8/41 (19.5 %)	5/19 (26.3 %)	3/22 (13.6 %)	0.436	0.442 (0.090–2.166)
*Candida* spp., no. (%)	6/41 (14.6 %)	2/19 (10.5 %)	4/22 (18.2 %)	0.668	1.889 (0.305–11.684)
Total number of pathogens, median (IQR)	2 (2–3)	2 (2–3)	2 (2–3.8)	0.795	1.006 (0.641–1.580)

**Table 1:** Continued.

	Descriptive analysis				Univariate analysis
	Total population	Favorable	Treatment	P value	OR (95 % CI)
	( n=48 )	outcome ( n=24 )	failure ( n=24 )		
Surgical management, no. (%)	39/48 (81.3 %)	15/24 (62.5 %)	24/24 (100 %)	0.002	NC
Debridement with metallic device retention, no. (%)	25/48 (52.1 %)	9/24 (37.5 %)	16/24 (66.7 %)	0.043	3.333 (1.020–10.898)
Complete metallic device exchange, no. (%)	1/48 (2.1 %)	0/24 (0 %)	1/24 (4.2 %)	>0.999	NC
Metallic device removal, no. (%)	6/48 (12.5 %)	3/24 (12.5 %)	3/24 (12.5 %)	>0.999	1.000 (0.181–5.533)
Flap removal, no. (%)	7/48 (14.6 %)	3/24 (12.5 %)	4/24 (16.7 %)	>0.999	1.400 (0.278–7.056)
Medical management					
ID referral, no. (%)	44/48 (91.7 %)	23/24 (95.8 %)	21/24 (87.5 %)	0.609	0.304 (0.029–3.157)
Delay from symptom onset to ID referral,	2.6 (0.1–13.2)	1.6 (0.0–4.1)	11.8 (0.3–19.9)	0.095	1.044 (0.992–1.099)
median (IQR), w					
≤2 w, no. (%)	33/48 (66.8 %)	20/24 (83.3 %)	13/24 (54.2 %)	0.060	0.236 (0.062–0.933)
Appropriate postoperative empirical	33/48 (68.8 %)	17/24 (68.8 %)	16/24 (66.7 %)	>0.999	0.824 (0.242–2.797)
antimicrobial therapy, no. (%)					
Parenteral treatment, no. (%)	45/48 (93.3 %)	23/24 (95.8 %)	22/24 (91.7 %)	>0.999	0.478 (0.040–5.658)
Duration of parenteral treatment, median (IQR), d	46.0 (27.0–84.0)	42.0 (28.5–84.5)	50.0 (27.3–77.8)	0.298	0.996 (0.984–1.009)
Switch for oral administration only, no. (%)	16/43 (37.2 %)	7/21 (33.3 %)	9/22 (40.9 %)	0.755	1.385 (0.399–4.800)
Total duration of antimicrobial therapy, median (IQR), d	93 (64.0–127.5)	93.0 (84.0–127.5)	88.5 (67.8–123.3)	0.773	1.000 (0.992–1.008)
Outcome					
Follow-up since surgery, median (IQR), months	18.0 (11.2–31.0)	10.7 (7.6–26.2)	22.4 (11.9–43.6)	0.773	n/a
CRP level 2 weeks after surgery, median (IQR), mg L ${}^{-1}$	11.4 (3.9–20.7)	7.0 (3.6–12.3)	14.7 (7.8–24.0)	0.104	1.066 (0.996–1.140)
CRP level <10 mg L ${}^{-1}$ 2 weeks after surgery	18/38 (47.4 %)	11/18 (61.1 %)	7/20 (33.0 %)	0.104	0.343 (0.092–1.283)
Flap removal for any reason, no. (%)	11/48 (22.9 %)	3/24 (12.5 %)	8/24 (33.3 %)	0.168	3.500 (0799–15.340)

95 % CI, 95 % confidence interval; ASA, American Society of Anesthesiologists; CoNS, coagulase-negative staphylococci; CRP, C-reactive protein; EAT, empirical antimicrobial therapy; ESBL, extended-spectrum betalactamase; GNB, Gram-negative bacilli; ID, infectious disease; IQR, interquartile range; MRCoNS, methicillin-resistant coagulase-negative staphylococci; MRSA, methicillin-resistant *Staphylococcus aureus*; n/a, not applicable; NC, not calculable; OR, odds ratio; WBC, white blood cell. ${}^{a}$ Calculated for 10 additional years. ${}^{b}$ Including 7 *Escherichia coli*, 5 *Proteus mirabilis*, 5 *Klebsiella pneumoniae*, 4 *Enterobacter cloacae*, 2 *Proteus vulgaris*, 2 *Citrobacter koseri*, 2 *Morganella morganii*, 1 *Halfnia alvei*, 1 *Citrobacter freundii*, and 1 *Klebsiella oxytoca*.

## 3 Results

### 3.1 Baseline patient characteristics

Forty-eight patients with mandibular OCF-associated osteomyelitis were included. Thirty of the 48 included patients (62.5 %) were male, with a median age of 60.5 (IQR, 52.4–66.6) years and a median modified Charlson co-morbidity index of 4 (IQR, 3–5).

Flap reconstruction was mostly performed for carcinoma ( n=27/48 ; 56.3 %), and 34 out of 41 patients (82.9 %) had previous neck irradiation. The major donor flap was fibula ( n=46/48 ; 95.8 %), with the two last patients having received clavi-pectoral and serrato-costal flaps. Cervical lymphadenectomy and tracheotomy were performed in 23/48 (47.9 %) and 24/48 (50.0 %) patients, respectively. Antimicrobial prophylaxis followed the French guidelines in 42 (97.7 %) of the 43 evaluable patients, mostly based on amoxicillin–clavulanate.

Patients and index surgery characteristics are summarized in Table 1.

### 3.2 Infection characteristics

OCF-related osteomyelitis was mostly early ( n=43/48 ; 89.6 %) and was considered acute in 22/48 (45.8 %) cases. Main symptoms are presented in Table 1. Fever was present in 17/47 (36.2 %) patients. The median maximum CRP level was 90.3 mg L ${}^{-1}$ (IQR, 34.2–158.5). Clinical symptoms were associated with radiological signs for infection in 75.0 % of cases ( n=33/44 ).

**Table 2:** Diagnostic values of superficial samples in comparison with deep surgical samples (gold standard).

	Gold-standard samples	Local samples	Sensitivity	Specificity	PPV	NPV
*Staphylococcus aureus*	5 (25 %)	2 (10 %)	0.2	0.93	0.5	0.78
CoNS	1 (5 %)	6 (30 %)	1	0.74	0.17	1
*Streptococcus* spp.	10 (50 %)	10 (50 %)	0.6	0.6	0.6	0.6
*Enterococcus* spp.	6 (30 %)	1 (5 %)	0.17	1	1	0.74
Enterobacteriaceae	11 (55 %)	5 (25 %)	0.45	1	1	0.6
Non-fermenting GNB	5 (25 %)	18 (90 %)	0.8	0.07	0.22	0.5
Anaerobes	2 (10 %)	4 (20 %)	0.5	0.83	0.25	0.94
*Candida* spp.	3 (15 %)	2 (10 %)	0.33	0.94	0.5	0.89
Unidentified oropharyngeal flora	2 (10 %)	2 (10 %)	0	0.89	0	0.89

CoNS, coagulase-negative staphylococci; GNB, Gram-negative bacilli; NPV, negative predictive value; PPV, positive predictive value.

Gold-standard surgical samples were collected in 41/48 (85.4 %) patients, with a median of three (IQR, 1–5) samples per patient, allowing for a microbiological documentation in 40/41 (97.6 %) cases. Only three documented infections were monomicrobial. Main implicated pathogens were Enterobacteriaceae ( n=25/41 ; 61.0 %), streptococci ( n=22/41 ; 53.7 %) with a majority of milleri group ( n=14/41 ; 34.1 %), *Staphylococcus aureus* ( n=10/41 ; 24.4 %), *Enterococcus faecalis* ( n= 8/41; 19.5 %), anaerobes ( n=8/41 ; 19.5 %), and non-fermenting GNB ( n=8/41 ; 19.5 %). Only one patient had positive blood cultures (*Actinomyces odontolyticus*). Note that results of preoperative superficial microbiological samples were available in 26/48 (54.2 %) patients. When comparing gold-standard and superficial sample culture results in the 20 (41.6 %) patients for whom both were available (Table 2), only four (20.0 %) were fully consistent. A 100 % predictive negative value was found for CoNS, only. Conversely, specificity and predictive positive values were good for Enterobacteriaceae and *Enterococcus faecalis*.

Histopathological analysis was performed in 24/48 cases (50.0 %) with histological signs of infection in 12/22 (54.5 %) cases including chronic inflammation in 8/22 (36.4 %).

Out of the eight patients with no gold-standard microbiological documentation, all had local clinical and radiological signs of infection; four patients had histologically proven osteomyelitis, and six patients had local bacteriological samples highlighting invasive pathogens (milleri group streptococci, Enterobacteriaceae, and/or *Pseudomonas aeruginosa*).

### 3.3 Infection management

Thirty-nine (81.3 %) of the 48 included patients benefited from surgery, mostly consisting of debridement with implant retention (DAIR; n=25/39 ; 64.1 %). Flap had to be removed in 7/39 (17.9 %) cases during the initial septic surgery.

Empirical antimicrobial therapy was given for 16.5 (IQR, 7.8–31.8) days and considered appropriate in 33/48 cases (68.8 %). The most commonly empirical regimen ( n=22/48 ; 45.8 %) was a combination of a broad-spectrum beta-lactam targeting non-fermenting GNB (piperacillin–tazobactam, cefepim, or carbapenem) with either vancomycin or daptomycin. Anaerobes were empirically targeted in 47/48 (97.9 %) patients. Four (8.3 %) out of 48 patients empirically received an antifungal drug. After empirical antimicrobial therapy, the antimicrobial therapy was adapted to the definite microbiological documentation, with a complete oral administration possible in 16/43 (37.2 %) patients, only. Total duration of antimicrobial therapy was 93 (IQR, 64.0–127.5) days. Antimicrobial therapy was driven by an infectious disease (ID) specialist in 44/48 (91.7 %) cases, with a mean delay from symptoms to referral of 2.6 (IQR, 0.1–13.2) weeks. ID specialist advice was taken before surgery in 18/39 (46.1 %) of the operated patients.

### 3.4 Outcome and determinants of treatment failure

In a median follow-up of 18 (IQR, 11.2–31.0) months, 24/48 (50.0 %) treatment failures were observed, corresponding to symptom persistence ( n=16/48 ; 33.3 %), relapse ( n=5/48 ; 10.4 %), requirement for additional surgical procedure ( n=20/48 ; 42.6 %) including 4/48 (8.3 %) flap removal, and 3/48 (6.3 %) infection-related death.

**Figure 1:**
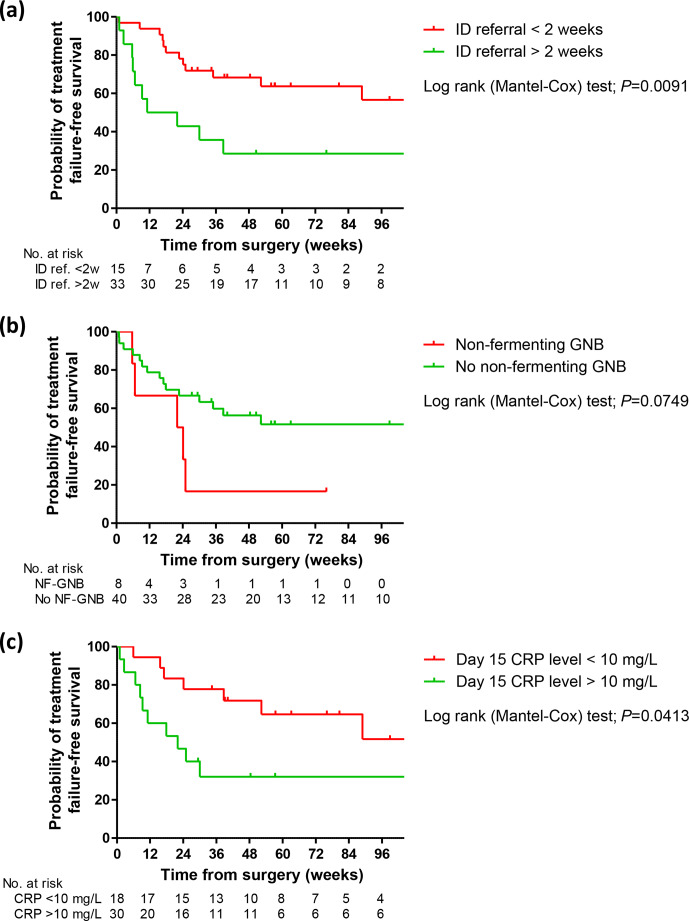
Kaplan–Meier curves showing the probability of failure-free survival according to early ID specialist referral **(a)**, non-fermenting GNB infection **(b)**, and normalization of the biological inflammatory syndrome **(c)**. ID, infectious disease; CRP, C-reactive protein; GNB, Gram-negative bacilli.

Baseline characteristics and infection diagnostic criteria were not different between the subsets of patients with or without treatment failure (Table 1). A higher proportion of patients treated with DAIR were observed in the treatment failure subset ( n=16/24 ; 66.7 % versus 9/24 ; 37.5 %; p=0.043 ), which consequently appeared as a determinant of treatment failure (OR, 3.333; 95 % CI, 1.020–10.898, p=0.046 ). The only other determinant of treatment outcome was an early referral to ID specialist (OR, 0.236 if ≤2 weeks; 95 % CI, 0.062–0.933; p=0.035 ). Non-fermenting GNB infections tended to be associated with a higher risk of failure (OR, 8.400; 95 % CI, 0.927–76.151; p=0.058 ) (Table 1). Kaplan–Meier curve analysis confirmed these results and additionally suggested the non-negative state of the biological inflammatory syndrome after 2 weeks as another potential predictor of treatment failure (Fig. 1).

Note that the eight patients with no gold-standard microbiological diagnosis received 92 (IQR, 69.8–112.0) days of antimicrobials, after a surgery in four patients. Persistent infection was noted in three patients, including two requiring an additional surgery and one death.

Concerning the nine patients who did not benefit from surgery, all had early postoperative infection. They all received an appropriate antimicrobial therapy, based on the result of multiple local samples, available for all patients, and guided by an infectious disease specialist, for a total duration of 98 (IQR, 86–127) days. No treatment failure was observed after a follow-up of 15.3 (IQR, 6.2–26.0) months after the end of treatment. Two patients died of non-infectious causes. No specificity could be highlighted in this specific subset of patients.

## 4 Discussion

OCF-related osteomyelitis represents an emerging BJI due to the increasing use of this surgical reconstruction technique, especially after head and neck cancer surgery. This infection can be considered complex, as it is mostly occurring in highly co-morbid patients and without consensual guidance for their difficult surgical management and broad-spectrum antimicrobial therapy to avoid treatment failure and flap loss.

We present here the first large series describing diagnosis, microbiological characteristics, management, and outcome of OCF-related osteomyelitis. Our results confirmed the poor prognosis of this infection, with a 50 % treatment failure rate despite complex medico-surgical support.

Diagnosis of OCF-related osteomyelitis is challenging and relies on an array of clinical, radiological, histological, and microbiological arguments. Clinical characteristics and infection chronology have to be known to avoid management delay. Even if the differential diagnosis with SSTI or early vascular complications can be difficult (Pohlenz et al., 2012), OCF-related osteomyelitis should be suspected in case of any incision incident including local inflammation, purulent discharge, nonunion or sinus tract, bone or device exposure, and abscess, as for other BJIs (Osmon et al., 2013). They mostly occur within 3 months post-surgery, consistent with previously published series, in which the superficial or deep nature of SSI and the existence of osteomyelitis were not stipulated (Durand et al., 2015; Lin et al., 2018). As expected for acute infections, bone abnormalities were not constant on imaging, with a third of patients presenting bone lysis only, but CT scan is also crucial to assess local complications (McCarty et al., 2019). Unfortunately, histopathological analysis was not available for all patients, impeding precise conclusions, but it might play an important role in doubtful cases as for other BJIs (Kapadia et al., 2016; Osmon et al., 2013; ICM, 2022).

Concerning microbiological documentation, the stringent definition used aimed to limit consideration of potential contaminants. Consistently with previously published series (Durand et al., 2015; Becker et al., 1978; Park et al., 2015), we highlighted a majority of polymicrobial infections, explained by intra-buccal exposure. As reported by Durand et al. (2015), a majority of GNB were surprisingly noted, along with the more expected streptococci, staphylococci, and anaerobes. Another questionable finding was the high proportion of enterococci and non-fermenting GNB. These microbiological considerations raise several issues. First, surgical-site contamination by local commensal flora might lead to polymicrobial infections, among which distinguishing sample contamination with saprophytic species from invasive isolates makes the microbiological diagnosis challenging The use of the IDSA (Infectious Diseases Society of America) guidelines for prosthetic joint infection diagnosis to interpret the microbiological result in the particular setting of head and neck surgery might not be appropriate (Osmon et al., 2013). Of note, as for other BJIs, is that sensitivity, specificity, and predictive values of superficial samples were insufficient to recommend them in the diagnostic strategy. Very few other studies analyzed the correlation of preoperative oral swabs and surgical deep samples in head and neck cancer SSI, and they did not demonstrate a good concordance either (Becker et al., 1978; Yang et al., 2013). Second, the high level of polymicrobism complicates the laboratory diagnostic process, leading to greater potential contaminants under the label “unidentified oropharyngeal flora”, which might underestimate the prevalence of some pathogens such as anaerobes, including *Actinomyces* spp. Third, the recommended antimicrobial prophylaxis based on amoxicillin–clavulanate might not be appropriate, and broader-spectrum molecules should be considered. Finally, this microbiological description advocates for the use of broad-spectrum empirical antimicrobial therapy awaiting definitive bacteriological diagnosis. A combination of piperacillin–tazobactam and daptomycin or cefepim with vancomycin and metronidazole could be adequate options to target GNB, streptococci, staphylococci, and anaerobes. Given the severity of the infection, this complex microbiological documentation, and its poor bone penetration, simple oral empirical antimicrobial therapy such as amoxicillin–clavulanate cannot be recommended.

OCF-related osteomyelitis was associated with a treatment failure rate of 50 %, and it was 22.9 % for flap loss. None of the baseline characteristics of patients and infection were found to influence clinical outcome in our series. Among these factors, prior radiotherapy could increase the risk of flap failure such as inducing fibrosis, damaging the microvasculature, or being associated with wound healing disorders (Schultze-Mosgau et al., 2002; Mueller and Schultze-Mosgau, 2009). However, if it represents a debated risk factor of SSI (Mücke et al., 2012; Bourget et al., 2011; Benatar et al., 2013; Halle et al., 2017), it was not associated with an increased risk of infection treatment failure. The impact of surgical strategy on treatment outcome has been poorly evaluated. In our series, DAIR had been highlighted with an increased risk of failure. However, the choice of surgical strategy in OCF-related osteomyelitis is challenged by the risk of vascular damage and flap loss in the early postoperative period. Unlike other device-associated infections, the benefit of complete foreign body removal has never been demonstrated and might not be required in most situations of early infections, except in the event of infection-induced flap loss. Similarly, antibiotic therapy features did not influence treatment outcome in our series. The appropriate total duration of antimicrobial therapy is unknown. Most included patients received a 3-month course of treatment, as recommended for other device-associated BJIs. Additionally, an early infectious disease specialist referral positively influences the outcome. This point highlights the importance of a multidisciplinary infection management with ear, nose, and throat surgeons and maxillofacial surgeons; microbiologists; radiologists; and ID specialists to help with infection diagnosis, determining optimal medico-surgical strategy, and limiting treatment failure. Such a dedicated trained team is required to personalize disease management, as highlighted for other complex BJI management by the dedicated French healthcare network (Ferry et al., 2019).

Some limitations of our study should be addressed. First, it includes the classical biases of retrospective and single-center design studies. Indeed, the long recruitment period and changes in practices and actors could make this series heterogeneous and our sample is relatively small. However, our series represents the first large description of this emerging complex infection and provides important insights into its management. Therefore, future investigations are needed to more precisely analyze the impact of different surgical strategies and medical management, even if larger sample size and prospective studies might be difficult to perform in this field. Second, the definition used for patient inclusion can be debated, as no consensual definition of OCF-associated osteomyelitis exists. We used a practical clinical point of view, with a mix of clinical, radiological, microbiological, and histological arguments, as none of the CDC surgical-site infection or other bone and joint infection definitions can be perfectly applied to this very specific entity. Finally, it was not possible to assess the main mechanism implicated in the infection occurrence, especially to differentiate direct inoculation from a consequence of microvascular issues of the flap, but both usually result in a severe postoperative infection. Patients with obvious early vascular failure were not included. Note that, in our center, rates of early failure (irreversible arterial or venous thrombosis ≤14 d), revision rate for anastomoses, and partial failure (i.e., loss of the skin paddle without the bony part of the flap) have been estimated at 6 %, 8 %, and 10 %, respectively.

## 5 Conclusions

Osteomyelitis following mandibular reconstruction with OCF represents difficult-to-treat infections with a high risk of treatment failure. Our results advocate for a multidisciplinary management, including an early ID specialist referral to manage the antimicrobial therapy driven by complex microbiological documentation, which might include a broad-spectrum empirical antimicrobial therapy targeting Gram-positive and Gram-negative bacteria, and anaerobes.

## Data Availability

Underlying research data can be accessed on request from the corresponding author.

## Team list

: Tristan Ferry; : Tristan Ferry, Florent Valour, Thomas Perpoint, Florence Ader, Sandrine Roux, Claire Triffault-Fillit, Agathe Becker, Anne Conrad, Cécile Pouderoux, Pierre Chauvelot, Paul Chabert, Johanna Lippman, Evelyne Braun, Marie Simon, Christian Chidiac; : Clémentine Daveau, Philippe Céruse, Carine Fuchsmann, Thais Mouton, Jacques Blanc, Arnaud Gleizal, Mathieu Daurade, Jérôme Bourlet, Pierre Breton, Antoine Pierrefeu, Nicolas Sigaux, Sébastien Lustig, Elvire Servien, Cécile Batailler, Stanislas Gunst, Axel Schmidt, Matthieu Malatray, Eliott Sappey-Marinier, Michel-Henry Fessy, Anthony Viste, Jean-Luc Besse, Philippe Chaudier, Lucie Louboutin, Quentin Ode, Adrien Van Haecke, Marcelle Mercier, Vincent Belgaid, Arnaud Walch, Sébastien Martres, Franck Trouillet, Cédric Barrey, Ali Mojallal, Sophie Brosset, Camille Hanriat, Hélène Person; : Frédéric Laurent, Céline Dupieux-Chabert, Laetitia Béraud, Camille Kolenda, Jérôme Josse, Tiphaine Roussel-Gaillard; : Caroline Pic, Faustine Bredeche, Christian Bauer, Clotilde Bettinger, Mikhail Dziadzko, Caroline Macabéo; : Fabien Craigheiro, Loic Boussel, Jean-Baptiste Pialat; : Isabelle Morelec, Marc Janier, Francesco Giammarile; : Michel Tod, Marie-Claude Gagnieu, Sylvain Goutelle; : Eugénie Mabrut.
